# Neonatal hyperoxia leads to white adipose tissue remodeling and susceptibility to hypercaloric diet

**DOI:** 10.14814/phy2.15769

**Published:** 2023-07-11

**Authors:** Alyson Deprez, Marie‐Amélie Lukaszewski, Coraline De Sousa Do Outeiro, Jéssica H. Poletto Bonetto, Ying He, Anik Cloutier, Daniela Ravizzoni Dartora, Anne Monique Nuyt

**Affiliations:** ^1^ Sainte Justine University Hospital (CHU Sainte‐Justine) and Research Centre, Department of Pediatrics, Faculty of Medicine Université de Montréal Montreal Quebec Canada; ^2^ Department of Pharmacology and Physiology, Faculty of Medicine Université de Montréal Montreal Quebec Canada

**Keywords:** developmental programming, hypercaloric diet, hyperoxia, inflammation, metabolism, preterm birth, white adipose tissue

## Abstract

Individuals born preterm are at higher risk of cardiovascular and metabolic diseases in adulthood, through mechanisms not completely understood. White adipose tissue in humans and rodents is a dynamic endocrine organ and a critical player in the regulation of metabolic homeostasis. However, the impact of preterm birth on white adipose tissue remains unknown. Using a well‐established rodent model of preterm birth‐related conditions in which newborn rats are exposed during postnatal days 3–10 to 80% of oxygen, we evaluated the impact of transient neonatal hyperoxia on adult perirenal white adipose tissue (pWAT) and liver. We further assessed the effect of a second hit with a high‐fat high‐fructose hypercaloric diet (HFFD). We evaluated 4‐month‐old adult male rats after 2 months of HFFD. Neonatal hyperoxia led to pWAT fibrosis and macrophage infiltration without modification in body weight, pWAT weight, or adipocyte size. In animals exposed to neonatal hyperoxia vs. room air control, HFFD resulted in adipocyte hypertrophy, lipid accumulation in the liver, and increased circulating triglycerides. Overall, preterm birth‐related conditions had long‐lasting effects on the composition and morphology of pWAT, along with a higher susceptibility to the deleterious impact of a hypercaloric diet. These changes suggest a developmental pathway to long‐term metabolic risk factors observed clinically in adults born preterm through programming of white adipose tissue.

## Graphical abstract

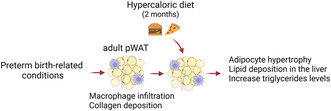

## 1 INTRODUCTION

Preterm birth (<37‐week gestation) accounts for nearly 10% of births worldwide. Scientific and technological advances in perinatal and neonatal care in the last three decades have substantially increased the survival of infants born preterm. With the growing number of babies born preterm reaching adulthood, long‐term health consequences are becoming apparent. Individuals born preterm are at higher risk of chronic cardiovascular (Crump et al., 2021; Crump, Howell, Stroustrup, et al., 2019; Hovi et al., 2016; Telles et al., 2020) and metabolic diseases (Crump et al., 2011; Crump et al., 2020a; Hofman et al., 2004; Hovi et al., 2007; Liao et al., 2020; Sipola‐Leppänen et al., 2015) as well as lipid metabolism disorders (Crump, Sundquist, & Sundquist, 2019). Evidence suggests that mechanistic pathways to chronic diseases differ from the general population and result from both perturbed organ development and (mal)adaptation to the neonatal prematurity conditions (Flahault et al., 2020). Preterm birth is characterized by a premature abrupt transition from intra to extrauterine life. This exposure to higher oxygen levels compared with *in utero* oxygen tension—that is often exacerbated by required neonatal oxygen supplementation—may result in oxidative stress (Davis & Auten, 2010) and inflammation during a critical period of organ system development and maturation (Nuyt et al., 2017).

We previously showed in a well‐established and clinically relevant rodent model mimicking preterm birth associated conditions that transient neonatal exposure to hyperoxia‐induced long‐term cardiovascular, renal, and muscular alterations (Deprez et al., 2021; O'Reilly & Thébaud, 2014; Ravizzoni Dartora et al., 2022; Yzydorczyk et al., 2008) associated with oxidative stress and inflammation (Bertagnolli et al., 2014; Mian et al., 2019). Indeed, transient neonatal rat exposure to high concentrations of oxygen mimics the combination of a sudden and sustained rise in partial pressure of oxygen (pO_2_) associated with birth in an immature organism (O'Reilly & Thébaud, 2014).

In general adult population, systemic oxidative stress and inflammation promote metabolic alterations that include white adipose tissue remodeling, adipocyte hypertrophy and fibrosis, and an altered secretome profile (Kaartinen et al., 2020; Unamuno et al., 2018). A similar profile is observed in animal models of metabolic disturbance and obesity (Ichioka et al., 2011; Sun et al., 2011). Thus, considering the relevance of white adipose tissue in the regulation of metabolic homeostasis and the detrimental environment associated with preterm birth, we hypothesize that transient neonatal hyperoxia leads to alterations in the white adipose tissue and lipid metabolism. We further hypothesize that neonatal hyperoxia predisposes to metabolic maladaptation to a hypercaloric diet in adulthood.

The current study therefore aimed to determine whether newborn rats exposed to transient hyperoxia experienced altered weight gain, white adipose tissue remodeling, systemic glucose and lipid metabolism perturbations, and hepatic lipid deposition in adulthood. The study also aimed to evaluate the response to a high‐fat high‐fructose diet (HFFD) in adulthood.

## 2 MATERIALS AND METHODS

### 2.1 Ethics statement

The study was approved by the Animal Ethics Committee of Sainte‐Justine Hospital (CHU Sainte‐Justine) in Montreal, Quebec, Canada. All animals were treated according to the *Guide to the Care and Use of Experimental Animals* from the Canadian Council on Animal Care, as per *Physiological reports* policy.

### 2.2 Animals

**FIGURE 1:**
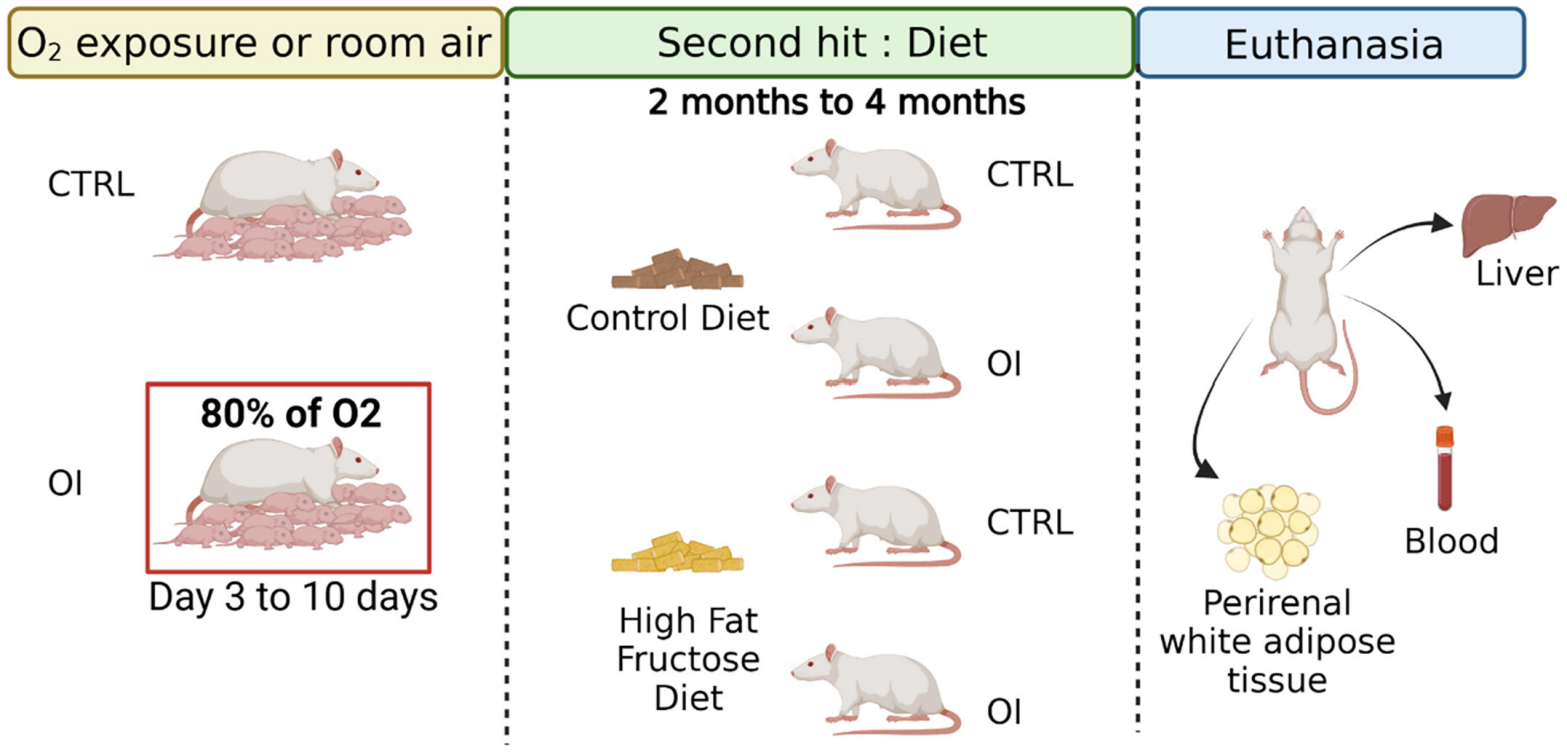
Experimental model and procedures. After a transient neonatal exposure to hyperoxia (OI), to mimic preterm birth‐related condition, or room air (CTRL) from 3 to 10 days of life, Sprague–Dawley rats were fed with a control diet or high fat fructose diet from 2 to 4 months. At 4 months, liver, perirenal white adipose tissue, and blood were collected.

At postnatal day 1 (P1), Sprague–Dawley litters (Charles River Laboratories, St. Constant, QC) were each culled to 12 pups with six pups/sex and maintained from P3 to P10 in either (1) 80% O_2_ (OxyCycler ProOx P110, BioSpherix) for the oxygen‐induced injury (OI) group, *n* = 14 L or (2) room air control (CTRL) group, *n* = 14 L, as previously described (Bertagnolli et al., 2014; Mian et al., 2019; Yzydorczyk et al., 2008; Figure 1). To prevent maternal morbidity associated with O_2_ toxicity, dams were interchanged every 12 h with a surrogate mother from a litter maintained at room air. At P10, the end of hyperoxia exposure, the litters kept in 80% of oxygen were transferred to room air until the end of the experiments. Control litters remained in room air with their dam (without interchange). We previously showed that dam interchange *per se* did not affect pup survival, growth, or blood pressure (Yzydorczyk et al., 2008). The animals were kept in standard housing conditions and weaned at 4 weeks. Litters were kept intact until weaning, that is, including males and females, since a reduction or sex imbalance in the number of pups could interfere with lactation and growth. After weaning, only male rats were included in the subsequent experiments. In rats, the perinatal period corresponds in many organs to a developmental stage equivalent to that of human infants born very preterm in the early third trimester of gestation (Bertagnolli, Luu, et al., 2016; Lewandowski & Leeson, 2014; O'Reilly & Thébaud, 2014).

### 2.3 Intervention: HFFD

At 4 weeks, CTRL and OI rats were transferred into individual cages with free access to water and the standard rodent chow: control diet (CD) from Teklad Global, Harlan Laboratories, USA (2018X Teklad Global 18% Protein Extruded Rodent Diet—3.1 kcal/g, containing 18.4% protein, 6.0% fat, and 44.2% carbohydrates). At 2 months of age (postpuberty and early adulthood), half of the rats from CTRL and OI were randomly assigned either to *ad libitum* high fat fructose diet, HFFD (Teklad Global, Harlan Laboratories, USA—4.5 kcal/g, containing 17% protein, 21% fat tallow, and 49% carbohydrates with 30.4% of fructose;) or remained with the CD, resulting into four experimental groups (*n* = 7 per group): CTRL, CTRL/HFFD, OI, and OI/HFFD (Figure 1). The HFFD was formulated to meet the criteria of our protocol (TD.140173), while the CD is the standard diet used in the animal facility of the CHU Sainte‐Justine Research Center. See comparative table (Table S1) of CD and HFFD macronutrients and nutrient profile.

### 2.4 Growth, food consumption, and glucose metabolism

Growth was assessed by body weight at postnatal days 1, 3 (immediately before hyperoxia exposure), 10 (end of hyperoxia exposure), and then weekly until the end of experiment at 4 months. Following the guidelines of the Canadian Council on Animal Care and the US National Institutes of Health Guide for the Care and Use of Laboratory Animals weekly (vs. daily), manipulation and cage‐enriched environment contribute to minimize stress in the rats. Starting at 2 months of age, food consumption was also recorded weekly up to 4 months of age. Food intake was measured by the delta weight of food once per week.

An oral glucose tolerance test (OGTT) was performed one week prior to the sacrifice at 4 months. Following an overnight 16‐hour fasting, rats (*n* = 6 per group) received an oral bolus administration of 50% glucose solution (2 g/kg; Sigma‐Aldrich). Blood glucose levels were assessed from jugular venous samples at times T0 (basal, prior to the gavage), 30, 60, and 120 min after glucose administration, using an automatic glucometer (OneTouch® Ultra, LifeScan).

### 2.5 Tissue samples

At 4 months, after an overnight fasting (16 h), rats were anesthetized by isoflurane inhalation (2–3% isoflurane/L O_2_), weighed, and decapitated. Trunk blood samples (*n* = 6–7 per group) were collected into K2‐EDTA coated tubes (BD Vacutainer), gently mixed, and centrifuged at 3000 g for 10 min at 4°C. Plasma aliquots were stored at −80°C until assayed.

For molecular analysis, the liver and perirenal white adipose tissue (pWAT) were rapidly removed, weighed, then snap‐frozen in liquid nitrogen, and stored at −80°C. For histology assessment, pWAT samples were fixed for 24 h in 4% paraformaldehyde in phosphate‐buffered saline solution, embedded in paraffin, and then sliced (12 μm) with a microtome for slide preparation. The liver was collected and embedded in Tissue‐Tek® optimal cutting temperature compound (O.C.T. compound, Sakura Finetek USA Inc.), froze on dry ice and sectioned into 7‐μm slices using a cryostat (ThermoFisher Scientific).

### 2.6 Leptin and lipid profile

Plasma levels of triglycerides, cholesterol, high‐density lipoprotein (HDL), and low‐density lipoprotein (LDL) were assessed in 4‐month‐old rats by the central biochemistry laboratory at CHU Sainte‐Justine. Leptin was measured using an enzyme‐linked immunosorbent assay (ELISA) kit (Abcam, ab100773), according to the manufacturer's instructions.

### 2.7 Histology and immunofluorescence

Adipocyte size was evaluated using hematoxylin and eosin staining (Sigma‐Aldrich). We measured adipocyte perimeter and area on four images randomly selected from three different areas of the pWAT sections (10X magnification Axio‐scan. Z1). We then quantified a minimum of 300 adipocytes per rat, using ImageJ software (NIH). We assessed collagen content with Sirius Red staining (kit Picrosirius Red 24,901–500, Polysciences) and visualized on 8–10 images of pWAT per rat at 10X magnification under polarized light, using a Leica DMi8 microscope (Leica Biosystems). The percentage of Sirius Red‐positive pixels over the total area was quantified using ImageJ software.

Prior to immunostaining, sections of pWAT were rehydrated by successive baths of xylene, 100%, 70%, and 50% alcohol solution, and tap water. For antigen retrieval, sections were incubated with trypsin solution (0.5%) at 37°C for 15 min. Tissues were incubated with primary antibodies overnight at 4°C, washed, and incubated with secondary fluorescent antibodies for 1 h at room temperature. We visualized the total number of macrophages in pWAT using immunofluorescence with rabbit anti‐rat CD68 antibody (Abcam, ab125212; dilution: 1/300) and a counterstaining with DAPI (stains cell nucleus), a Leica DMi 8 microscope, and ImageJ. Each section was examined at 40X magnification, and 10 images were randomly acquired. The number of labeled cells was counted, and the surface area of each image was quantified to express the data as number of cells/mm^2^.

LipidTOX green neutral lipid stain reagent (Invitrogen, Thermo Fisher Scientific, Toronto, Canada, H34475) was used to quantify hepatic lipid deposition. For LipidTOX staining, 8–10 images per rat at 40X magnification were acquired with the Leica SP8 confocal microscope (Leica Microsystems) and green‐positive pixels area over the total tissue area were counted using ImageJ software and expressed relative to the number of cells (DAPI) identified on the image.

### 2.8 Extraction of RNA from pWAT and quantitative real‐time polymerase chain reaction (RT‐qPCR)

Total RNA was extracted from pWAT homogenized samples (Omni Bead Ruptor 24, PerkinElmer, Inc.) using the RNeasy Lipid Tissue Mini Kit (Qiagen Inc), according to the manufacturer's instructions. The extraction was followed by a DNAse digestion step to remove any contaminating genomic DNA. Then, the RNA was quantified using the CLARIOstar Reader (BMG Labtech), and 1 μg of RNA from each sample was removed for reverse‐transcription using the QuantiTect Reverse Transcription Kit (Qiagen Inc).

RT‐qPCR was performed in a reaction mix containing SensiFAST SYBR No‐ROX Kit (Bioline), forward and reverse primers (400 nM of each), and cDNA sample. Primer efficiency was calculated from a 1:10 serial dilution standard curve. Samples were done in technical duplicate, and all reactions were normalized by the expression of *GAPDH* housekeeping gene (see Table 1). Reactions were performed under the following conditions: polymerase activation at 95°C for 2 min, followed by forty cycles at 95°C for 5 s for denaturation, 65°C for 10 s for annealing, and 72°C for 15 s for elongation. Data were expressed as cycle threshold (ΔCT) values, where ΔCT = CT_GAPDH_ – CT_gene of interest_, normalized to the CTRL/CD group. Expression levels of the genes of interest were calculated using the 2‐^ΔΔ^Cq method (Livak & Schmittgen, 2001). The quality of the RT‐qPCR analysis was confirmed by the presence of a single melt‐curve peak, representing a single amplification product.

**TABLE 1:** Primers used in RT‐qPCR.^a^

Gene name	Gene bank number	Primer sequence (5′‐3′)	Product size (bp)
*Gapdh*	NM_017008.4	Forward GGACCTCATGGCCTACATGG Reverse ATTCGAGAGAAGGGAGGGCT	206
*Il 6*	NM_012589.2	Forward GCCCACCAGGAACGAAAGTC Reverse TGGCTGGAAGTCTCTTGCGG	80
*Il1b*	NM_031512.2	Forward CAGCTATGGCAACTGTCCCT Reverse AACAGGTCATTCTCCTCACTGT	70
*Mcp1*	NM_031530.1	Forward TTAATGCCCCACTCACCTGC Reverse TTGAGCTTGGTGACAAATACTACA	130
*Tgfb1*	NM_021578.2	Forward GACCGCAACAACGCAATCTA Reverse TTCCGTCTCCTTGGTTCAGC	298
*Tnfα*	NM_012675.3	Forward ACTGAACTTCGGGGTGATCG Reverse CCGCTTGGTGGTTTGCTACG	155

^a^ RT‐qPCR, quantitative reverse‐transcription polymerase chain reaction.

### 2.9 Statistical analysis

Results were expressed as mean ± SEM. For data evaluation, we used two‐way ANOVA, considering exposure to hyperoxia and diet as factors (unless otherwise indicated), followed by multiple comparisons where applicable. When significant ANOVA results were obtained, we performed a post hoc Tukey's honest significant difference test. All statistical analyses were performed using GraphPad Prism 6.0 (GraphPad Software). Significance was set at *p* < 0.05.

## 3 RESULTS

### 3.1 Body weight, food intake, and glucose metabolism

**FIGURE 2:**
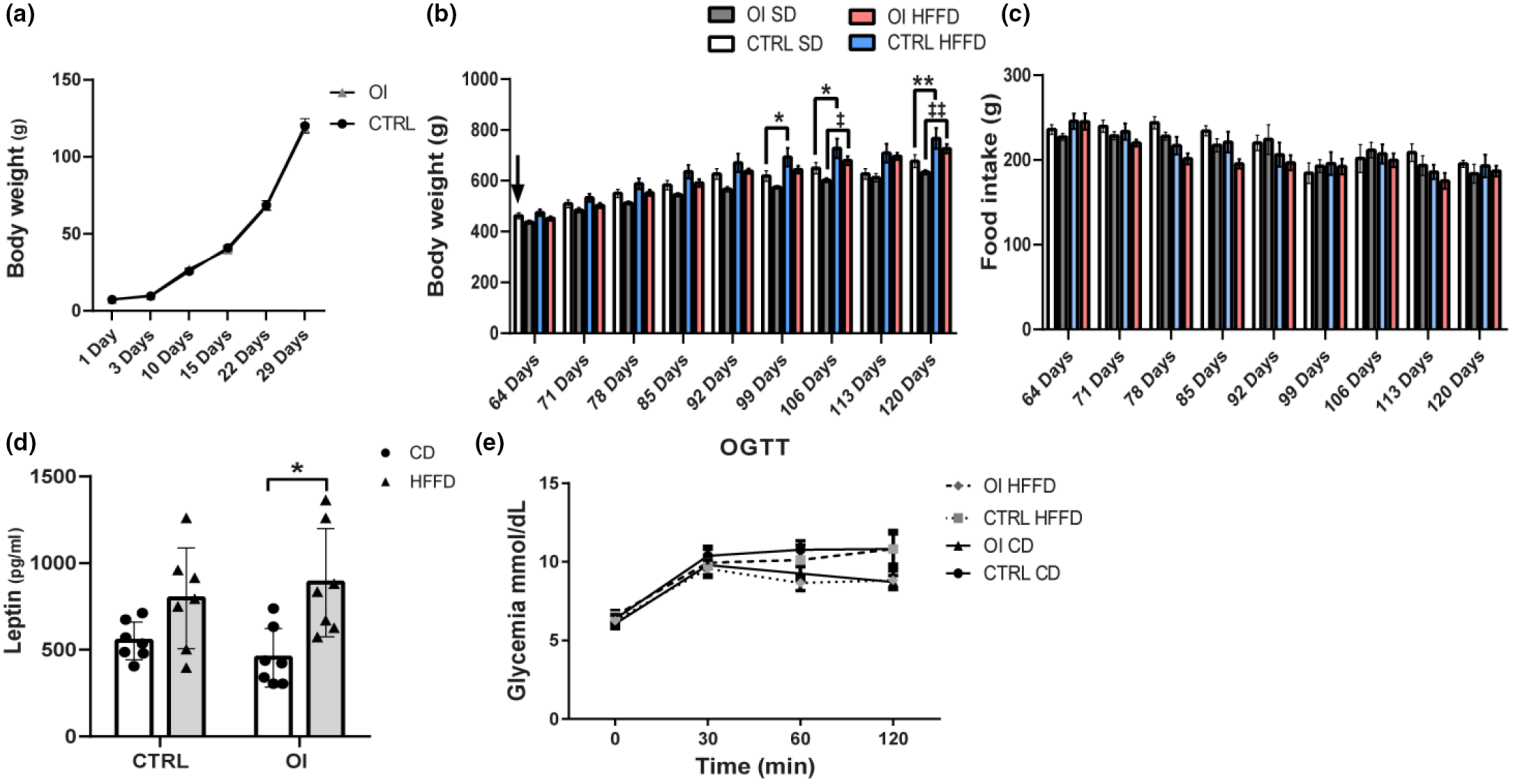
Body weight, food intake, leptin, and oral glucose tolerance test (a) Body weight from 1 to 29 days of life and (b) from 64 days (2 months) to 120 days (4 months) of life from control (CTRL) and oxygen‐induced injury (OI) with control diet (CD) or high fat fructose diet (HFFD). (c) Food intake per rat per week from 64 days to 120 days of life from CTRL and OI with CD or HFFD. (d) Leptin levels at 2 months from CTRL and OI with CD or HFFD. (E) At 120 days, animals from CTRL and OI with CD or HFFD were administered by gavage 2 g/kg of body weight of a 50% glucose solution. Blood glucose levels were evaluated immediately prior to the gavage (basal, 0 minutes) and then 30‐, 60‐, and 120‐minute postglucose administration. Error bars represent means ± SEM; *n* = 6–7 per group. Statistical analyses were performed using student *t*‐test (a) or two‐way ANOVA with Tukey's post‐test, (b, c) *p CTRL/CD versus CTRL/HFFD, ‡*p* OI/CD versus OI/HFFD, * or ^‡^ *p* < 0.05; ** or ^‡‡^ *p* < 0.01; *** or ^‡‡‡^ *p* < 0.001. (d) *p < 0.05 versus group indicated. Black arrow in panel (b) indicates the initiation of HFFD diet.

In the first month of life, there was no difference in body weight between OI and CTRL rats (Figure 2a). Starting at age 2 months, both HFFD groups gained more weight overall than rats with the control diet (Figure 2b), despite the fact that the food intake did not change (Figure 2c).

At age 4 months, serum leptin levels were similar between CTRL and OI with control diet. In response to HFFD, serum leptin levels increased significantly in OI but not in CTRL (Figure 2d). Glycemic responses to an oral glucose tolerance test were not different between the four groups (Figure 2e).

### 3.2 Remodeling of the pWAT

**FIGURE 3:**
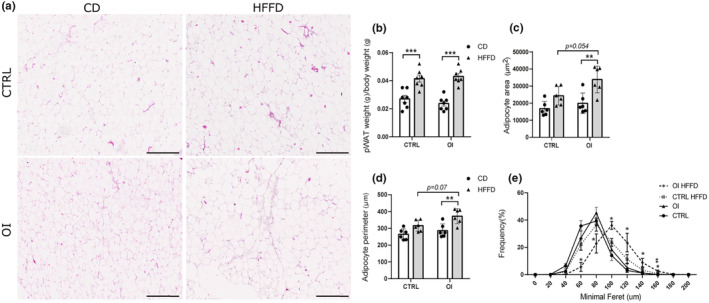
Adipocyte morphometric assessment (a) Representative images of hematoxylin and eosin coloration of perirenal white adipose tissue (pWAT) from control (CTRL) and oxygen‐induced injury (OI) with control diet (CD) or high fat fructose diet (HFFD). (b) pWAT weight to body weight from CTRL and OI with CD or HFFD. (c) Evaluation of the area (per μm^2^), (d) perimeter (μm) and (e) minimal Feret diameter (μm) of adipocytes in pWAT from CTRL and OI with CD or HFFD. Error bars represent means ± SEM; *n* = 7 per group. Scale bar = 246.8 μm. Statistical analyses were performed using two‐way ANOVA with Tukey's post‐test, (b–d) **p < 0.01; ***p < 0.001 versus group indicated. (e) *p OI/CD versus OI/HFFD and ^‡^ *p* CTRL/HFFD versus OI/HFFD, * or ^‡^ *p* < 0.05.

At age 4 months, pWAT mass relative to body weight was similar between CTRL and OI with control diet. After 2 months of HFFD, pWAT mass relative to body weight increased significantly and similarly in both OI and CTRL without the effect of the neonatal hyperoxia exposure (Figure 3b). Adipocyte area and perimeters were similar between CTRL and OI with control diet. In response to HFFD, adipocyte area and perimeter increased significantly in OI, not in CTRL (Figure 3c and d). Similarly, the frequency distribution of adipocyte size (assessed by minimal Feret diameter) was significantly shifted toward larger sizes only in OI/HFFD compared with all other groups (Figure 3a, e).

**FIGURE 4:**
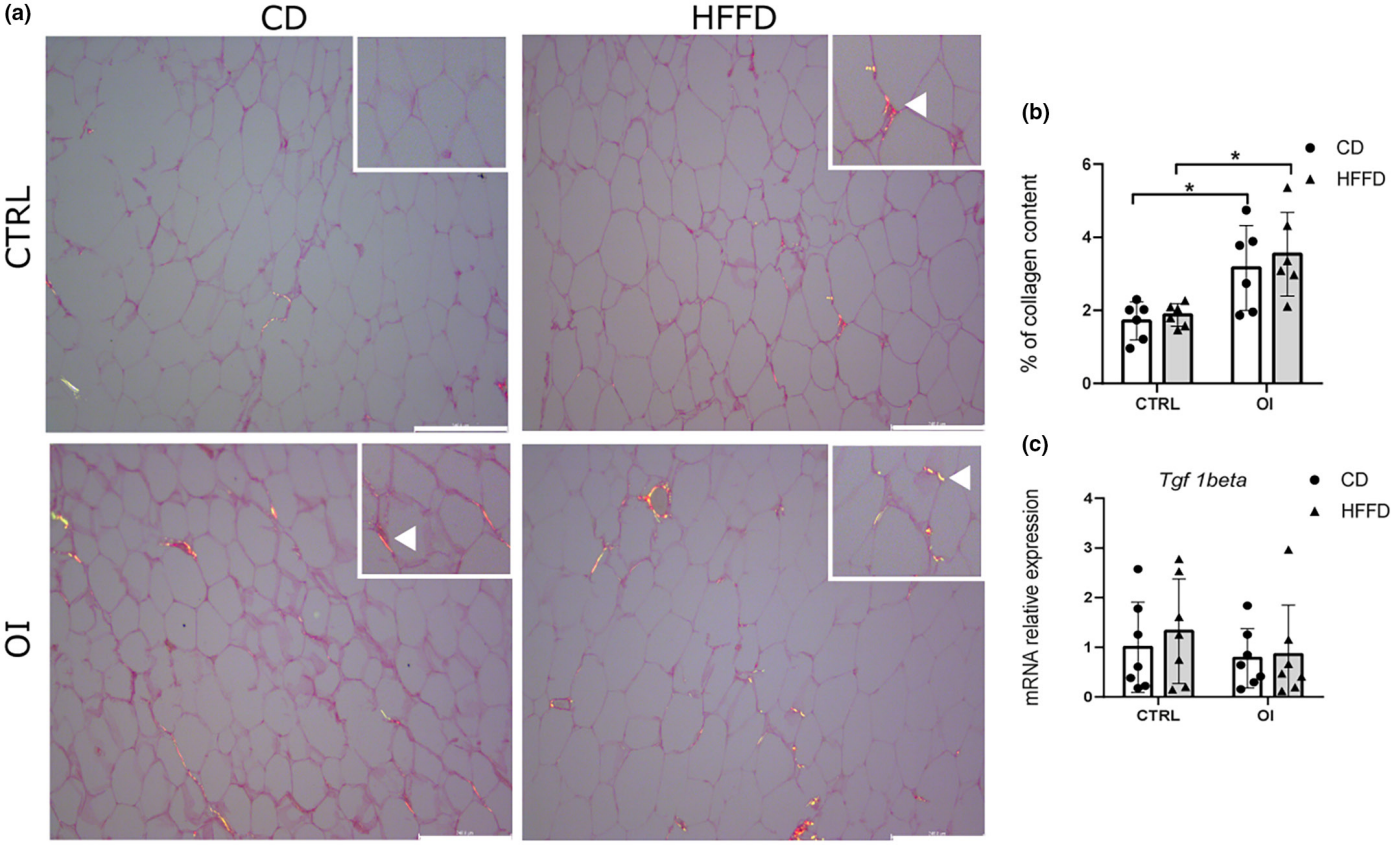
Fibrosis in adipose tissue. (a) Representative images of Sirius Red coloration (collagen type I) in perirenal white adipose tissue (pWAT) sections from control (CTRL) and oxygen‐induced injury (OI) with control diet (CD) or high fat fructose diet (HFFD). (b) Quantification of Sirius Red staining (percentage of red pixels/area) and (c) mRNA relative expression of Tgf1 beta from CTRL and OI with CD or HFFD. Error bars represent means ± SEM; *n* = 6–7 per group. Scale bar = 246.8 μm. Statistical analyses were performed using two‐way ANOVA with Tukey's post‐test, *p < 0.05. versus group indicated.

To assess the presence of fibrosis in pWAT, we quantified collagen content in histological sections. Collagen content was significantly higher in OI vs. CTRL with control diet. Nevertheless, in response to HFFD, no further change was observed in either group while the collagen content remained significantly increased in OI/HFFD vs. CTRL/HFFD (Figure 4a, b).

Considering the ubiquitous role of transforming growth factor beta 1 (TGFB1) in adipose tissue fibrosis (Rajangam et al., 2016), we assessed tgfb1 gene expression in pWAT. No difference in tgfb1 gene was observed between experimental groups (Figure 4c).

### 3.3 Inflammatory markers in pWAT

**FIGURE 5:**
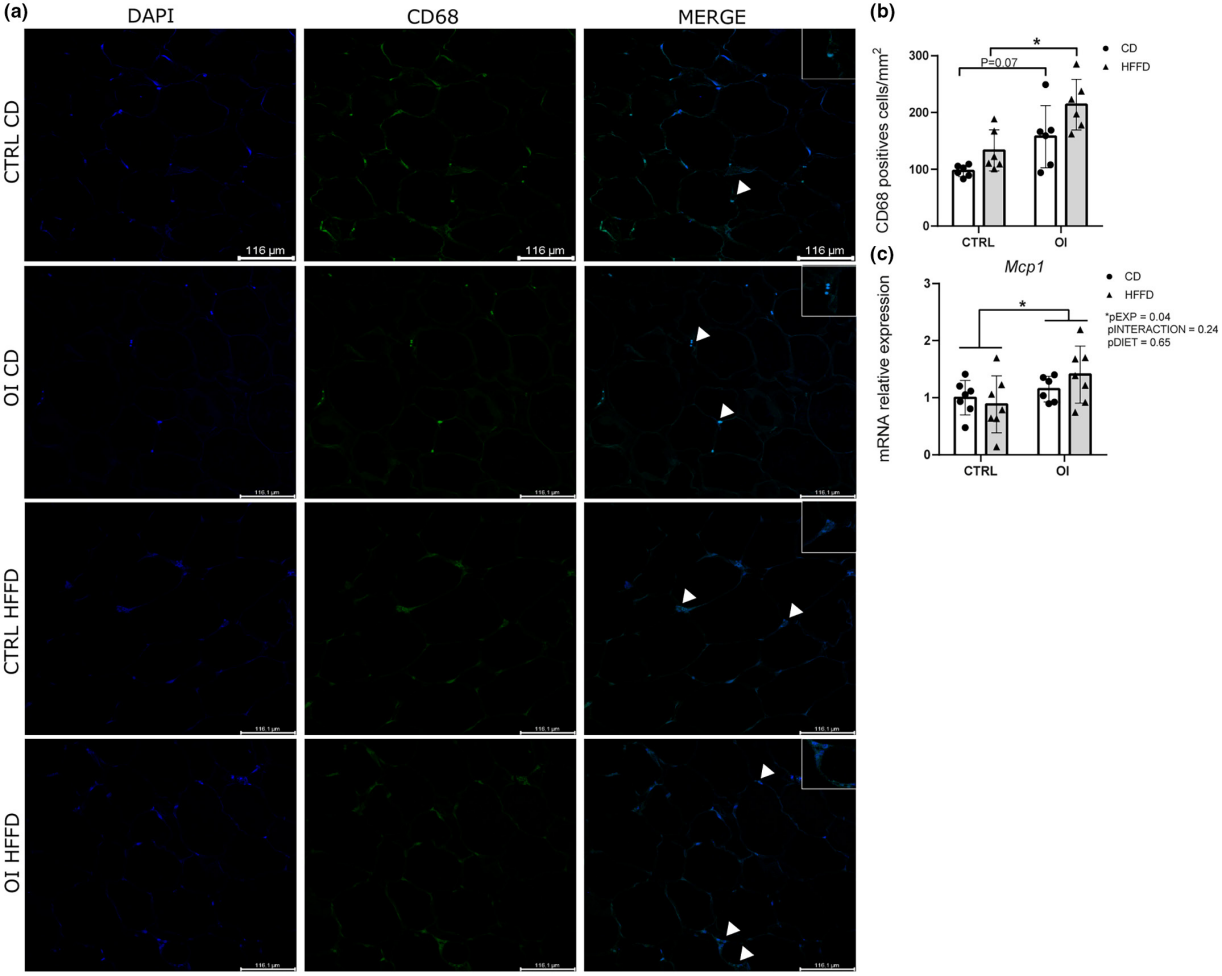
Macrophages and proinflammatory markers. (a) Representative images of CD68 (pan‐macrophage marker, green) and DAPI (nuclei marker, blue) and merge of CD68 and DAPI on perirenal white adipose tissue (pWAT) sections from control (CTRL) and oxygen‐induced injury (OI) with control diet (CD) or high fat fructose diet (HFFD). White arrows identify macrophages. (b) Quantification of the number of CD68+ cells per mm^2^ in pWAT from CTRL and OI with CD or HFFD. (c) mRNA relative expression of Mcp1 in pWAT from CTRL and OI with CD or HFFD. Error bars represent means ± SEM; *n* = 6–7 per group. Scale bar = 116.1 μm. Statistical analyses were performed using two‐way ANOVA with Tukey's post‐test, *p < 0.05 versus group indicated. LSM = least square means.

Preterm birth‐related conditions are associated with systemic inflammation (Luu et al., 2016; Soto‐Rivera et al., 2015), which in turn could mediate adipocyte hypertrophy and fibrosis (Sun et al., 2011). To evaluate adipose tissue inflammation, we measured pan‐macrophage density and proinflammatory markers in pWAT. OI with control diet showed a slight increase in the density of CD68+ cells (*p* = 0.07) versus CTRL with control diet; in response to HFFD, macrophage density was significantly higher in OI/HFFD vs. CTRL/HFFD (Figure 5a, b).

Dysfunctional adipocytes and macrophage release proinflammatory monocyte chemoattractant protein‐1 (MCP‐1; Christiansen et al., 2005; Gerhardt et al., 2001; Sartipy & Loskutoff, 2003). We found overall upregulation of *Mcp1* gene expression in OI vs CTRL rats, corroborating the increased presence of macrophages in pWAT in OI rats although its expression remained unchanged by HFFD (Figure 5c). We complemented the evaluation of proinflammatory cytokines in pWAT by measuring the gene expression of *Tnfα*, *Il1b*, and *Il6*; no differences between groups were observed (Figure S1).

### 3.4 Hepatic alterations

**FIGURE 6:**
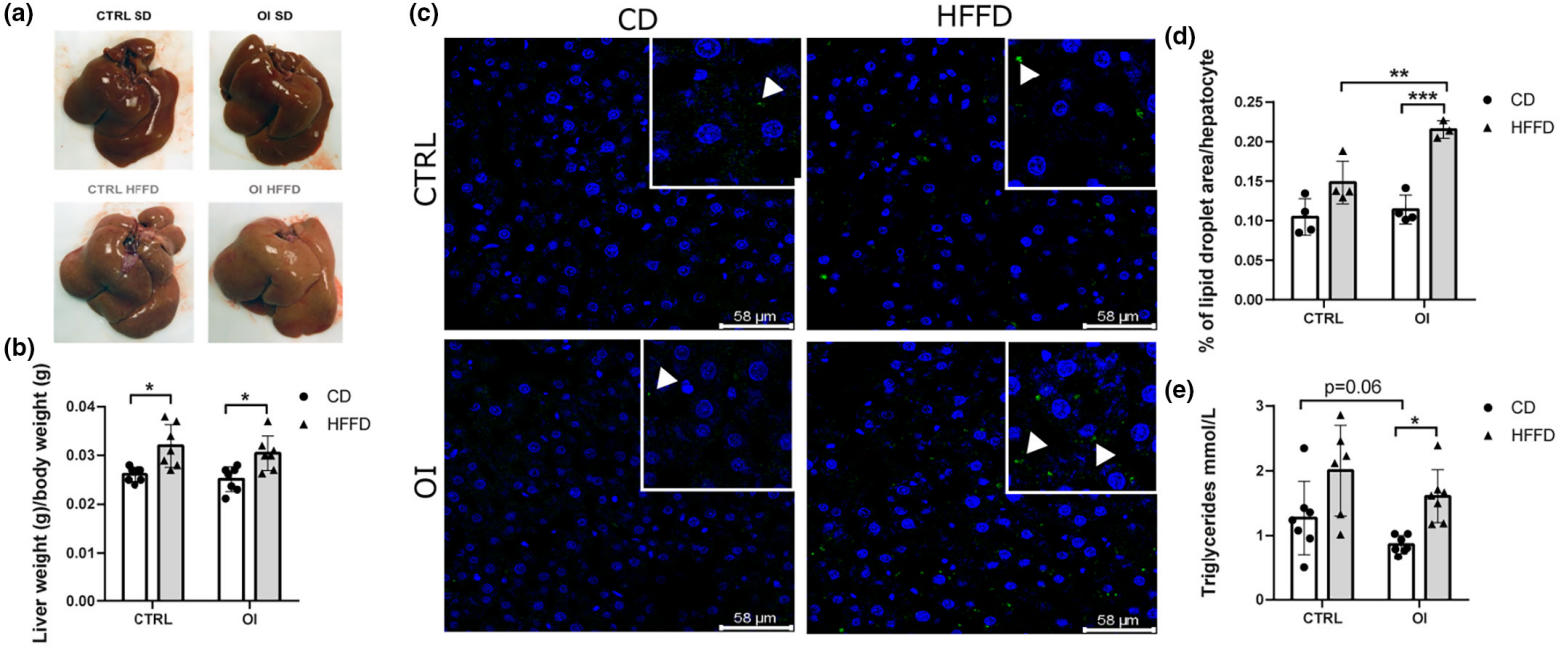
Lipid deposition in the liver. (a) Pictures of the liver from control (CTRL) and oxygen‐induced exposure (OI) with control diet (CD) or high fat fructose diet (HFFD). (b) Liver weight to body weight from CTRL and OI with CD or HFFD (c) Representative images of lipidTox staining (neutral lipid marker, green) and DAPI (nuclei marker, blue) of liver sections from males CTRL OI with CD or HFFD. In the magnified insert, white arrows identify lipid droplets. (d) Quantification of the percentage of lipid droplet area per hepatocyte and (e) triglyceride levels from blood from CTRL and OI with CD or HFFD. Error bars represent means ± SEM; (b, e) *n* = 6–7 per group and (d) *n* = 3–4 per group. Statistical analyses were performed using two‐way ANOVA with Tukey's post‐test, *p < 0.05; **p < 0.01; ***p < 0.001 versus group indicated.

To determine whether adipose tissue alterations were associated with liver modifications, we first measured total liver weight to body weight ratio, which were similar between CTRL and OI with control diet. In response to HFFD, liver‐to‐body weight ratio increased significantly and similarly in both OI and CTRL without the effect of the hyperoxia exposure (Figure 6a, b). Area covered by lipid droplets within the hepatocytes was similar between CTRL and OI with control diet. However, in response to HFFD, lipid droplet area significantly increased in OI only (Figure 6c, d).

We further examined whether alterations of hepatic lipid deposition had repercussions on systemic lipid profile. Serum triglyceride levels were slightly lower (*p* = 0.06) in OI as compared to CTRL with control diet, while HFFD led to a significant increase only observed in the OI group (Figure 6e). No alterations in cholesterol, HDL, and LDL levels were found between groups (Figure 2c, d).

## 4 DISCUSSION

We showed that transient neonatal exposure to hyperoxia leads to fibrosis and macrophage infiltration in pWAT in adulthood, despite similar body and pWAT weight. When challenged with a second hit, a hypercaloric diet, OI male rats vs. CTRL displayed more pronounced pWAT remodeling, adipocyte hypertrophy, hepatic lipid accumulation, and higher systemic triglyceride level. These results suggest that neonatal transient hyperoxia affects the capability to manage challenging metabolic environment later in life, which could contribute to the long‐term cardiovascular and metabolic consequences observed clinically in adults born preterm (Luu et al., 2016).

The concept of “developmental origins of health and disease (DOHaD)” suggests that intrauterine and perinatal environment has a determinant role in health and disease later in life (Barker & Martyn, 1992), including metabolic disorders. In humans, preterm birth occurs at a critical period of organogenesis and fetal maturation, with both acute and long effects on organ function. Indeed, clinical studies in adolescents and adults born preterm showed higher risk of cardiovascular disease, type 2 diabetes, and lipid metabolism disorders (Crump, Sundquist, & Sundquist, 2019). Interestingly, the current diet protocol did not impact glucose tolerance in either group, which might be related to the duration of the diet exposure, or to the postpuberty initiation of the diet, that is, after key developmental susceptibility period. It is well established that the white adipose tissue, an endocrine organ crucial to metabolic homeostasis (Symonds et al., 2012), develops in utero and matures throughout the lifespan (Spalding et al., 2008). At the end of the second trimester of gestation, the proportion of white adipose tissue from the fetal body weight increases progressively from 1% to 15% (Harding, 1971) in preparation to the upcoming transition to extrauterine life. In term born infant, mature adipose tissues are the main source of energy for thermal, nutritional, and ventilatory adaptation to the transition to postnatal life (Symonds et al., 2011).

Our findings show that neonatal hyperoxia exposure leads to pWAT remodeling with significant fibrosis, which is in accordance with enhanced fibrosis reported in the lungs, heart, and skeletal muscles in this experimental model (Bertagnolli, Dios, et al., 2016; Chen et al., 2007; Deprez et al., 2021). Taken together, these studies suggest that tissue fibrosis may be a common manifestation of tissue injury following transient neonatal hyperoxia. In chronic obesity, fibrosis driven by collagen deposition is a major contributor to adipose tissue dysfunction (Crewe et al., 2017). In humans as well as in animal models, fibrosis is associated with highly deleterious effects (DeBari & Abbott, 2020) and limited adipocyte physiological adaptation capacity (Divoux et al., 2010; Muir et al., 2016). In agreement with the latter, we found that OI rats challenged with a hypercaloric diet show increased pWAT adipocyte area, perimeter, and size. Different fat depots have specificities and distinct functions and accordingly could have different responses to the HFFD. pWAT is a visceral adipose depot with a well‐established relationship with metabolic disease (Chusyd et al., 2016), and it is a strong predictor of cardiovascular risk profile (Lee et al., 2018), including hypertension and coronary heart disease (Liu et al., 2019). Individuals born preterm are at higher risk of these cardiometabolic complications. Transcriptomic analysis of white adipose tissue depots has also revealed that pWAT is particularly susceptible to adverse early life conditions (Ahmad et al., 2021). Taken together, current findings have important implications considering that in obesity, adipocyte hypertrophy, especially from the visceral fat compartment, constitutes an independent risk factor for cardiometabolic diseases (Laforest et al., 2015). Overall, fibrosis may thus be a precursor to the morphological modifications in pWAT, impairing the capacity to further adapt to a metabolic challenge (second hit) with impaired lipid metabolism as suggested by increased triglycerides and fat infiltration in hepatocytes.

Increased density of the pan‐macrophage marker CD68 and upregulation of *Mcp1* gene expression, a monocyte chemokine, in OI rats compared with CTRL are also in favor of WAT remodeling. Indeed, under pathological conditions, adipose tissue remodeling is characterized and accelerated by immune cell infiltration (DeBari & Abbott, 2020; Hotamisligil et al., 1993). Expansion of macrophage density was also previously observed in the same model in adulthood and in males cardiac left ventricle (Mian et al., 2019), skeletal muscle (Deprez et al., 2021). Low‐grade chronic inflammation can induce macrophage infiltration mediated by the release of MCP1 from resident adipocytes (Kanda et al., 2006; Sartipy & Loskutoff, 2003; Weisberg et al., 2006). Thus, local inflammation (Crewe et al., 2017; Sun et al., 2011) could contribute to modulate tissue and organ alterations and to heightened risk of adult‐onset disease after preterm birth. We did not find any significant differences in the expression of proinflammatory cytokines TNF*α*, IL*β*, or IL6 although the high variability in gene expression of these cytokines within groups might have precluded clear conclusions. Of note, obesity can be characterized by a wide spectrum of heterogenic metabolic alterations (Mayoral et al., 2020; Neeland et al., 2018). A more adaptive role of white adipose tissue fibrosis in preventing the excessive enlargement of adipocytes by promoting a more rigid extracellular microenvironment has been speculated (Datta et al., 2018). This would result in sustained metabolic homeostasis so that not all obesity phenotypes show the same degree of adipose tissue fibrosis and inflammation.

Adipose tissue fibrosis contributes to the accumulation of lipids in other tissues such as the liver (Chait & den Hartigh, 2020). We observed increased liver weight, intrahepatic lipid deposition, and systemic triglycerides after a challenge with the hypercaloric diet only in the OI group. In studies of preterm young adults, lipid profile is mostly unaltered (Crump, Sundquist, & Sundquist, 2019; Flahault et al., 2020; Hovi et al., 2013), unless associated with increased BMI (Lewandowski et al., 2013). In addition, it was observed that in preterm individual adipose tissue deposit distribution is different compared with the general population such as ectopic deposition in liver or muscle (Louise et al., 2012). Interestingly, a recent population‐based cohort study (Constance) reports an increased incidence of nonalcoholic fatty liver disease in adults born preterm (Amadou et al., 2022).

In the general population, classical pathway to the metabolic syndrome involves initially adipocyte hypertrophy and the release of proinflammatory cytokines and adipokines, secondarily leading to adipose tissue macrophage infiltration, fibrosis, and lipid deposition in other organs (de Zegher et al., 2009; Sun et al., 2011). However, in our model, transient neonatal hyperoxia led to pWAT macrophage infiltration and fibrosis without adipocyte hypertrophy. This pWAT remodeling of OI rats was associated with enhanced adipocyte hypertrophy and lipid infiltration in the liver, despite similar increase in pWAT weight in response to the hypercaloric diet, suggesting that a different pathway to metabolic disturbance could be present after preterm birth. White adipose tissue impairments characterized by the macrophage infiltration, fibrosis, and lipid deposition even in the absence of hypertrophy could be a key factor mediating metabolic and cardiovascular disease risk in preterm population (Goossens, 2008).

This study has limitations that merit comment and alternative explanations to consider. Although extensively used to study short‐ and long‐term complications of preterm birth (Deprez et al., 2021; O'Reilly & Thébaud, 2014; Yzydorczyk et al., 2008), transient exposure to neonatal hyperoxia does not fully capture its complexity. For instance, prematurity‐associated complications and treatments such as parenteral nutrition, corticosteroids, and impaired postnatal growth, can also contribute to disrupt postnatal organ growth of the preterm infant. Second, this first study examined only male rats and results cannot be extrapolated to females. Juvenile and adult impact of preterm birth and, experimentally, of neonatal high oxygen exposure is similar for males and females for a number of cardiometabolic outcomes but not for all (Crump et al., 2020a, 2020b; Crump, Howell, Stroustrup, et al., 2019; Crump, Sundquist, Winkleby, & Sundquist, 2019; Popescu et al., 2013; Yzydorczyk et al., 2008). Furthermore, it is important to examine separately females’ metabolic outcomes, considering the adipose tissue distribution is sex‐specific, including in neonates and that sexual hormones are associated with metabolism (Hedrington & Davis, 2015; Kur et al., 2020) and therefore could be differentially modulated by neonatal conditions (Symonds et al., 2012). Lastly, assessment of earlier stages of development in the OI model is needed to identify optimal time windows for early intervention or preventive approaches.

In summary, these findings contribute to our understanding of the impact of preterm birth on white adipose tissue and its capacity to adapt to a hypercaloric diet later on. Adipose tissue plays a crucial role in the adaptation to extrauterine life, in metabolic homeostasis, and conversely in cardiometabolic disease. The pathophysiological pathways we observed with inflammation and fibrosis without adipocyte hypertrophy seemed to differ from the classical model of obesity‐related metabolism disorder. Determining the role of white adipose tissue remodeling in the development of cardiometabolic diseases associated with prematurity will advance the quest for optimal preventive and therapeutic strategies.

## AUTHOR CONTRIBUTIONS

AD, MAL, DRD, and AMN contributed to the conception or design of the work. AD, MAL, CDS, JHBP, YH, and AC contributed to the acquisition of data for the work. AD, MAL, CDS, and JHBP contributed to the analysis of data for the work. AD, MAL, DRD, and AMN contributed to the interpretation of data in the work. AD and MAL contributed to drafting the work. DRD and AMN contributed to revising the work.

All authors approved the final version of the manuscript and agreed to be accountable for all aspects of the work in ensuring that questions related to the accuracy or integrity of any part of the work are appropriately investigated and resolved.

## FUNDING INFORMATION

AD was supported by fellowships of the FRQNT (*Fonds de recherche du Québec—Nature et Technologies*, 275929). MAL was supported by fellowships of the Molly Towell Perinatal Research Foundation, the Canadian Institute of Health Research (CIHR) Quebec Training Network in Perinatal Research and the CHU Sainte‐Justine Foundation. DRD was awarded fellowships from the *Société Québecoise d'Hypertension Artérielle*, the Canadian Vascular Network and the FRQS (*Fonds de recherche du Québec*—*Santé*, process number: 268142 and 36412). CDS was supported by a *Bourse de recrutement* (graduate studies award) from the Biomedical Sciences program at the Université de Montréal. JHPB was awarded fellowships from CIHR, the *Société Québecoise d'Hypertension Artérielle*, the CHU Sainte‐Justine Foundation and the FRQS Research Network in Perinatal Determinants of Children Health. Studies were supported by grants from CIHR, Canadian Foundation of Innovation and the Heart and Stroke Foundation of Canada (all to AMN). AMN was supported by the Cercle de Sainte‐Justine DOHaD Research Chair and a Tier 1 Canada Research Chair in Prematurity and Developmental Origins of Cardiovascular Health and Diseases.

## CONFLICT OF INTEREST STATEMENT

The authors have declared that they have no conflict of interest.

## Supporting information


**Figure S1:** Proinflammatory cytokine gene expression. mRNA relative expression of (a) *Tnfα*, (b) *Il1Beta*, and (c) *Il6* from control (CTRL) and oxygen‐induced exposure (OI) with control diet (CD) or high fat fructose diet (HFFD). Error bars represent means ± SEM; *n* = 6–7 per group.
**Figure S2:** Liver and lipid profile. (a) Number of hepatocytes per mm^2^ from control (CTRL) and oxygen‐induced injury (OI) with control diet (CD) or high fat fructose diet (HFFD). (b) Cholesterol, (c) LDL (low‐density lipoprotein), and (d) HDL (high‐density lipoprotein) levels from blood of CTRL and OI with CD or HFFD. Error bars represent means ± SEM; *n* = 7 per group, *n* = 3–4 per group for (a). Statistical analyses were performed using two‐way ANOVA with Tukey’s post‐test, *p < 0.05.
**Table S1:** Comparison table of the macronutrients between standard diet and high fat fructose diet (HFFD).

## Data Availability

Data are available for sharing upon reasonable request to the corresponding authors.
