# Supplementary material for: Neonatal hyperoxia leads to white adipose tissue remodeling and susceptibility to hypercaloric diet
Source: Physiol Rep. 2023 Jul 11;11(13):e15769. doi: 10.14814/phy2.15769 (PMC10336028; doi:10.14814/phy2.15769)
Supplement: Supplementary file 1 — Figure S1: Proinflammatory cytokine gene expression. mRNA relative expression of (a) Tnfα, (b) Il1Beta, and (c) Il6 from control (CTRL) and oxygen‐induced exposure (OI) with control diet (CD) or high fat fructose diet (HFFD). Error bars represent means ± SEM; n = 6–7 per group. Figure S2: Liver and lipid profile. (a) Number of hepatocytes per mm2 from control (CTRL) and oxygen‐induced injury (OI) with control diet (CD) or high fat fructose diet (HFFD). (b) Cholesterol, (c) LDL (low‐density lipoprotein), and (d) HDL (high‐density lipoprotein) levels from blood of CTRL and OI with CD or HFFD. Error bars represent means ± SEM; n = 7 per group, n = 3–4 per group for (a). Statistical analyses were performed using two‐way ANOVA with Tukey’s post‐test, *p < 0.05. Table S1: Comparison table of the macronutrients between standard diet and high fat fructose diet (HFFD). [file PHY2-11-e15769-s001.docx]

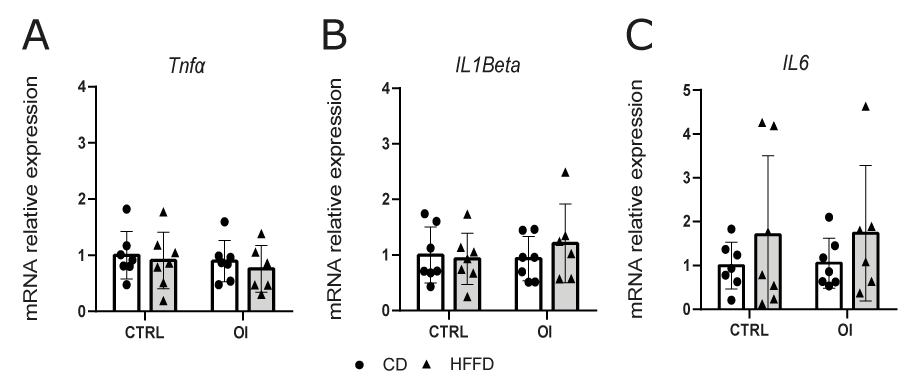


**Supplemental Figure 1: Proinflammatory cytokines gene expression.** mRNA relative expression of (A) *Tnfα*, (B) *Il1Beta* and (C) *Il6* from control (CTRL) and oxygen-induced exposure (OI) with control diet (CD) or high fat fructose diet (HFFD). Error bars represent means ± SEM; n=6-7 per group.


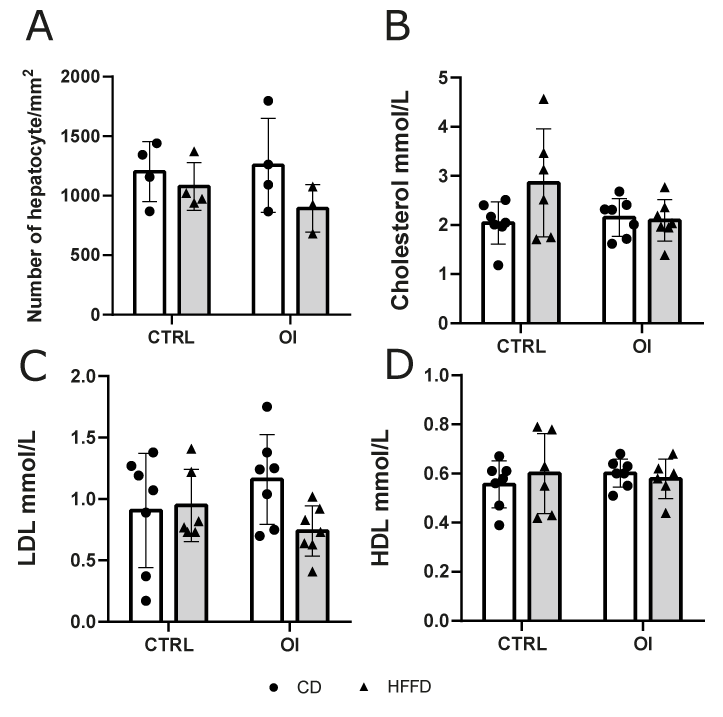


**Supplemental Figure 2: Liver and lipid profile.** (A) Number of hepatocytes per mm^2^ from control (CTRL) and oxygen-induced injury (OI) with control diet (CD) or high fat fructose diet (HFFD). (B) Cholesterol (C) LDL (Low Density Lipoprotein) and (D) HDL (High Density Lipoprotein) levels from blood of CTRL and OI with CD or HFFD. Error bars represent means ± SEM; n=7 per group, n=3-4 per group for (A). Statistical analyses were performed using two-way ANOVA with Tukey’s post-test, *p<0.05.

| Energy Density, Kcal/g | Standard diet | HFFD |
| --- | --- | --- |
|  | 3.1 | 4.5 |
| Macronutrients, % by weight |  |  |
| Protein | 18.4 | 17.3 |
| Carbohydrate | 44.2 | 48.5 |
| Fat | 6.0 | 21.2 |
| Saturated fatty acids (%) | 0.9 | 54 |
| Monounsaturated fatty acids (%) | 1.3 | 43 |
| Polyunsaturated fatty acids (%) | 3.4 | 3 |

**Supplemental table 1: Comparison table of the macronutrients between standard diet and high fat fructose diet (HFFD).**
